# A novel method, gel immersion endoscopic injection sclerotherapy, may make the procedure easier and more accurate

**DOI:** 10.1055/a-2114-0131

**Published:** 2023-07-27

**Authors:** Noriaki Sugawara, Taro Iwatsubo, Hironori Tanaka, Akitoshi Hakoda, Kazuhiro Ota, Toshihisa Takeuchi, Hiroki Nishikawa

**Affiliations:** Second Department of Internal Medicine, Osaka Medical and Pharmaceutical University, Osaka, Japan

Esophageal varices are caused by portal hypertension such as cirrhosis. Ruptured esophageal varices cause massive bleeding; therefore, preventive hemostasis is important. The standard procedures to prevent bleeding include endoscopic variceal ligation (EVL) and endoscopic injection sclerotherapy (EIS). EIS is a more difficult procedure than EVL but results in a lower recurrence rate and is more curative. In particular, problems are often faced in the process of inserting a needle into esophageal varices and injecting sclerosing agents into the vessel. Gel immersion endoscopy is a useful method for securing a visual field 1 . Additionally, a lower level of intraluminal pressure and wall tension is maintained 2 and the vessel lumen is kept thicker and wider than that during gas emersion ( Fig. 1 ). Therefore, EIS with a “Viscoclear” gel (Otsuka Pharmaceutical Factory, Tokushima, Japan) may allow easier needle insertion and sclerosing material injection into esophageal varices.

**Fig. 1:**
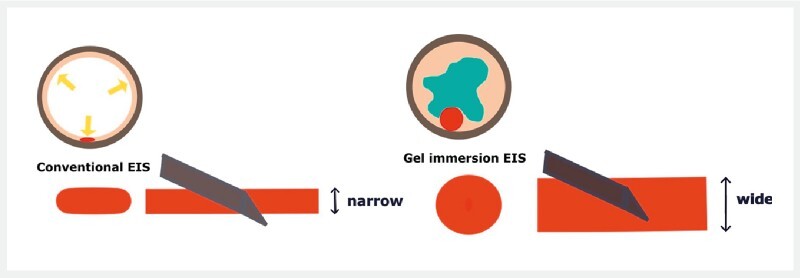
Gel immersion allows for lower levels of intraluminal pressure and maintenance of wall tension, while the vessel lumen is kept wider than during gas emersion.

Herein, a 73-year-old man was admitted to a psychiatric hospital for schizophrenia. Upper gastrointestinal endoscopy revealed F2 or larger esophageal varices that required treatment, and he was transferred to our hospital for prophylactic hemostasis ( Video 1 ). We performed EIS of the esophageal varices using gel immersion endoscopy. Under the gel, the vessels were thicker due to the lower pressure in the esophageal lumen ( Fig. 2 ), and ultrasound endoscopy confirmed that the vascular lumen remained circular ( Fig. 3 ). We were able to puncture the vessel under the gel and inject sufficient sclerosing agent. A total of 600 mL of gel was used and the procedure time was 4 min. No irrigation accessories were used. Gel-immersion EIS may be useful as it makes the puncture process easier and more accurate.

**Video 1** Gel-immersion endoscopic injection sclerotherapy (EIS) may be useful because it makes the puncture process easier and more accurate.


**Fig. 2:**
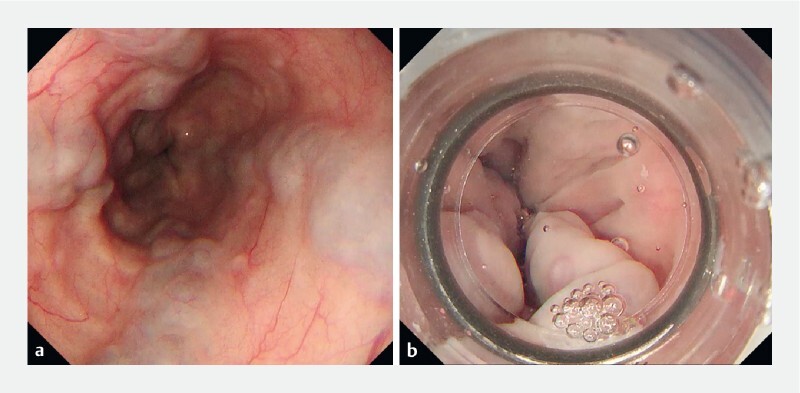
Endoscopic images showing esophageal varices. **a** Gas immersion endoscopy showing multiple esophageal varices. **b** Gel immersion endoscopy showing the varices as widely dilated.

**Fig. 3:**
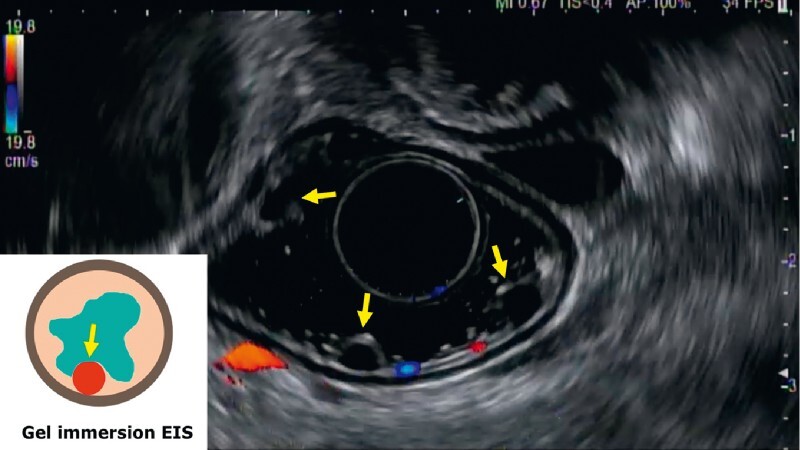
The endoscopic ultrasonography image and schema. Under the gel, the blood vessels present a circular shape and preserve the lumen.

Endoscopy_UCTN_Code_CCL_1AB_2AC_3AG

## Notes

Endoscopy E-Videos

https://eref.thieme.de/e-videos
*E-Videos* is an open access online section of the journal *Endoscopy* , reporting on interesting cases and new techniques in gastroenterological endoscopy. All papers include a high-quality video and are published with a Creative Commons CC-BY license. Endoscopy E-Videos qualify for HINARI discounts and waivers and eligibility is automatically checked during the submission process. We grant 100% waivers to articles whose corresponding authors are based in Group A countries and 50% waivers to those who are based in Group B countries as classified by Research4Life (see: https://www.research4life.org/access/eligibility/ ). This section has its own submission website at https://mc.manuscriptcentral.com/e-videos

## References

[1] Yano T Takezawa T Hashimoto K Gel immersion endoscopy: innovation in securing the visual field – clinical experience with 265 consecutive procedures Endosc Int Open 2021 9 E1123 E1127 34222638 10.1055/a-1400-8289 PMC8216780

[2] Yano K Yano T Nagayama M Hemostasis of an actively bleeding lesion at the ileocecal valve by low-pressure endoscopy using the gel immersion technique. Video-GIE 2021 VideoGIE 2021 6 184 186 33898898 10.1016/j.vgie.2020.11.019 PMC8058511

